# Identification of c-Met as a novel target of γ-glutamylcyclotransferase

**DOI:** 10.1038/s41598-023-39093-7

**Published:** 2023-07-24

**Authors:** Yumiko Saito, Keiko Taniguchi, Hiromi Ii, Mano Horinaka, Susumu Kageyama, Susumu Nakata, Osamu Ukimura, Toshiyuki Sakai

**Affiliations:** 1 Department of Drug Discovery Medicine, Kyoto Prefectural University of Medicine, Kajii-cho 465, Kawaramachi-Hirokoji Kamigyo-ku, Kyoto, 602-8566 Japan; 2 Department of Urology, Kyoto Prefectural University of Medicine, Kyoto, Japan; 3 Department of Clinical Oncology, Kyoto Pharmaceutical University, Kyoto, Japan; 4 Department of Urology, Shiga University of Medical Science, Shiga, Japan

**Keywords:** Molecular biology, Cancer, Oncogenes

## Abstract

γ-Glutamylcyclotransferase (GGCT) is highly expressed in multiple types of cancer tissues and its knockdown suppresses the growth of cancer cells in vitro and in vivo. Although GGCT is a promising target for cancer therapy, the mechanisms underlying the antitumor effects remain unclear. The knockdown of GGCT inhibited the MEK-ERK pathway, and activated the tumor suppressor retinoblastoma gene (*RB*) at the protein level in cancer cell lines. c-Met was down-regulated by the knockdown of GGCT in cancer cells and its overexpression attenuated the dephosphorylation of RB and cell cycle arrest induced by the knockdown of GGCT in lung cancer A549 cells. STAT3 is a transcription factor that induces c-Met expression. STAT3 phosphorylation and its nuclear expression level were decreased in GGCT-depleted A549 and prostate cancer PC3 cells. The simultaneous knockdown of AMPK and GGCT restored the down-regulated expression of c-Met, and attenuated the dephosphorylation of STAT3 and MEK-ERK-RB induced by the knockdown of GGCT in PC3 cells. An intraperitoneal injection of a GGCT inhibitor decreased c-Met protein expression in a mouse xenograft model of PC3 cells. These results suggest that the knockdown of GGCT activates the RB protein by inhibiting the STAT3-c-Met-MEK-ERK pathway via AMPK activation.

## Introduction

γ-Glutamylcyclotransferase (GGCT) was identified by a proteomic analysis as a protein that is expressed at a higher level in bladder cancer tissues than in normal tissues^1,2^. GGCT was thereafter shown to be highly expressed in various types of cancer tissues^3^, and higher GGCT expression levels correlates with a poor prognosis in breast cancer patients^4^, ovarian cancer^5^, and papillary thyroid cancer^6^. The overexpression of GGCT promoted the growth of NIH3T3 mouse fibroblasts^2^ while its knockdown inhibited that of multiple cancer cell lines^7^. Furthermore, the depletion of GGCT using siRNAs, lentiviral short hairpin RNAs, and antisense oligonucleotides targeting GGCT inhibited tumor growth in multiple tumor-bearing mouse models^5,8–10^.

The retinoblastoma gene (*RB*) was identified as the first tumor suppressor gene^11^, and is frequently inactivated in various malignant tumors at the protein level^12^. Complexes of cyclins and cyclin-dependent kinases (CDKs) phosphorylate and inactivate the RB protein. Inactivated RB releases E2F, and free E2F is crucial for cell cycle progression from the G1 phase to S phase as a transcription factor^12^. We recently reported that the knockdown of GGCT suppressed the mitogen-activated protein kinase (MAPK) pathway, such as MEK and ERK, in multiple cancer cell lines^13^. In this study, we found that the knockdown of GGCT enhanced the protein activity of RB.

We previously demonstrated that the knockdown of GGCT suppressed aerobic glycolysis, one of the hallmarks of cancer, and decreased the ratio of AMP to ATP, resulting in the activation of AMP-activated protein kinase (AMPK)^13^. AMPK is activated in response to cellular nutritional deficiency and phosphorylates multiple downstream factors, thereby coordinating autophagy, cellular metabolism, and cell growth^14^. AMPK activated by low nutrients was shown to inactivate mTOR complex 1 (mTORC1) by phosphorylating the regulatory-associated protein of mTOR, thereby inhibiting cell growth^15^.

To clarify the downstream molecules of GGCT-AMPK, we performed a DNA microarray analysis and found that GGCT up-regulated the expression of c-Met, also known as the hepatocyte growth factor (HGF) receptor. c-Met overexpression was previously reported to promote tumorigenesis and cancer cell growth in various malignancies^16,17^. To the best of our knowledge, this is the first study to show that c-Met is a novel downstream signal of GGCT that inactivates the RB protein.

## Results

### The knockdown of GGCT inactivates the MEK-ERK pathway and activates the RB protein

**Figure 1:**
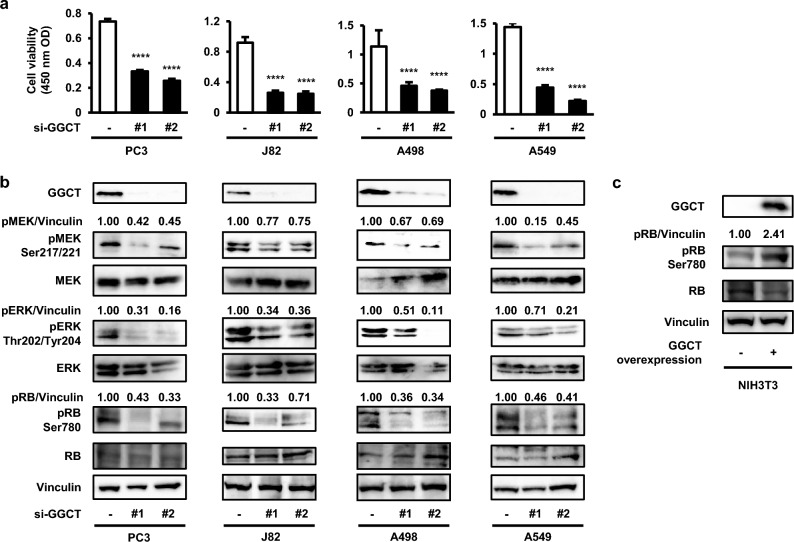
The knockdown of GGCT inhibits the MEK-ERK pathway and activates the RB protein. (**a**) The viability of PC3, J82, A498, and A549 cells treated with control siRNA or the indicated GGCT siRNAs for 120 h was examined by the CCK-8 assay. A one-way ANOVA with Dunnett’s test was used (versus control, N = 5 per group, ****p < 0.0001). The error bars represent the S. D. (**b**) A Western blot analysis of GGCT, pMEK Ser217/221, pERK Thr202/Tyr204, pRB Ser780, and their non-phosphorylated forms in PC3, J82, A498, and A549 cells treated with control siRNA or the indicated GGCT siRNAs for 72 h. Vinculin is shown as a loading control. All full-length blots are presented in Supplementary Fig. S4. (**c**) A Western blot analysis of GGCT, pRB Ser780, and its non-phosphorylated form in control or GGCT-overexpressing NIH3T3. Vinculin is shown as a loading control. All full-length blots are presented in Supplementary Fig. S4.

Since the depletion of GGCT was shown to induce growth inhibition and cell cycle arrest in various cancer cell lines^3^, we hypothesized that its knockdown may activate the RB protein. To confirm that GGCT contributes to proliferation in various types of cancers, we initially showed that the knockdown of GGCT inhibited the growth of prostate PC3, bladder J82, renal A498, and lung A549 cancer cells (Fig. 1a). MEK, ERK, and RB phosphorylation levels were decreased in GGCT-depleted cells (Fig. 1b). Conversely, RB phosphorylation levels were increased in GGCT-overexpressing NIH3T3 cells (Fig. 1c), which is consistent with our previous findings showing increases in MEK-ERK phosphorylation levels in GGCT-overexpressing NIH3T3 cells^13^. These results indicate that the knockdown of GGCT inactivated the MEK-ERK pathway and activated the RB protein.

### The knockdown of GGCT down-regulates c-Met expression

**Figure 2:**
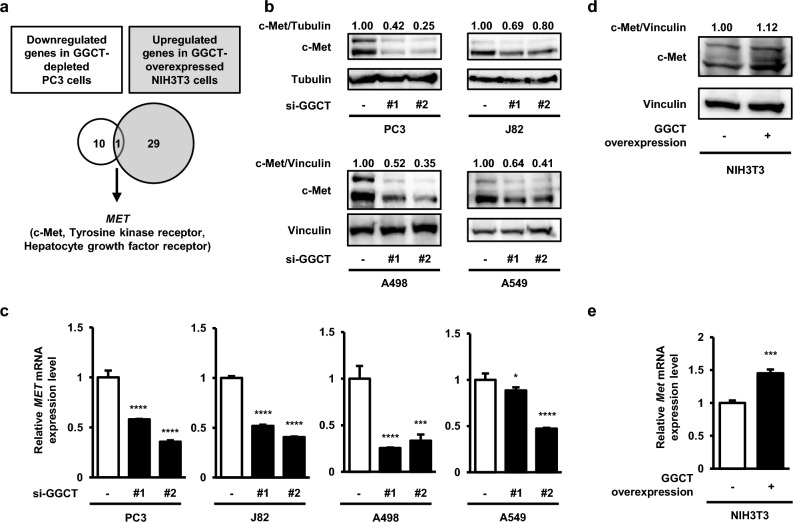
The knockdown of GGCT down-regulates c-Met. (**a**) A Venn diagram for the overlap between down-regulated genes in GGCT-depleted PC3 cells and up-regulated genes in GGCT-overexpressing NIH3T3 cells. (**b**) A Western blot analysis of c-Met in PC3, J82, A498, and A549 cells treated with control siRNA or the indicated GGCT siRNAs for 72 h. Tubulin or vinculin is shown as a loading control. All full-length blots are presented in Supplementary Fig. S5. (**c**) The expression of human-*MET* mRNA, as assessed by qRT-PCR, in PC3, J82, A498, and A549 cells at 72 h post-transfection with control siRNA or the indicated GGCT siRNAs. A one-way ANOVA with Dunnett’s test was used (N = 3 per group, *p < 0.05, ***p < 0.001, ****p < 0.0001). The error bars represent the S. D. (**d**) A Western blot analysis of c-Met in control or GGCT-overexpressing NIH3T3 cells. Vinculin is shown as a loading control. All full-length blots are presented in Supplementary Fig. S5. (**e**) The expression of mouse-*Met* mRNA, as assessed by qRT-PCR, in control and GGCT-overexpressing NIH3T3 cells cultured for 48 h. A two-tailed Student’s *t*-test was used (N = 3 per group, ***p < 0.001). The error bars represent the S. D.

Since the knockdown of GGCT inactivated the MEK-ERK pathway and activated the RB protein, we hypothesized that GGCT may regulate upstream factors of the MEK-ERK-RB pathway. To elucidate these upstream factors, we performed a transcriptome analysis using DNA microarrays. Among the 284 established receptor tyrosine kinase and growth factor genes, *MET* was the only gene significantly down-regulated in GGCT-depleted PC3 cells and significantly up-regulated in GGCT-overexpressing NIH3T3 fibroblasts (Fig. 2a). No evident phenotypic change was observed in GGCT-overexpressing cancer cells, we therefore concluded that the function of GGCT is saturated in cancer cells basically expressing abundant GGCT. Thus, we performed transfection of GGCT pCX4bsr vector in NIH3T3 cells with low expression of GGCT. c-Met mRNA and protein expression levels were verified by Western blot and qRT-PCR analyses. The knockdown of GGCT down-regulated c-Met protein and mRNA expression in PC3, J82, A498, and A549 cells (Fig. 2b,c). Conversely, c-Met protein and mRNA expression was up-regulated in GGCT-overexpressing NIH3T3 cells (Fig. 2d,e).

### The knockdown of GGCT activates the RB protein by down-regulating c-Met

**Figure 3:**
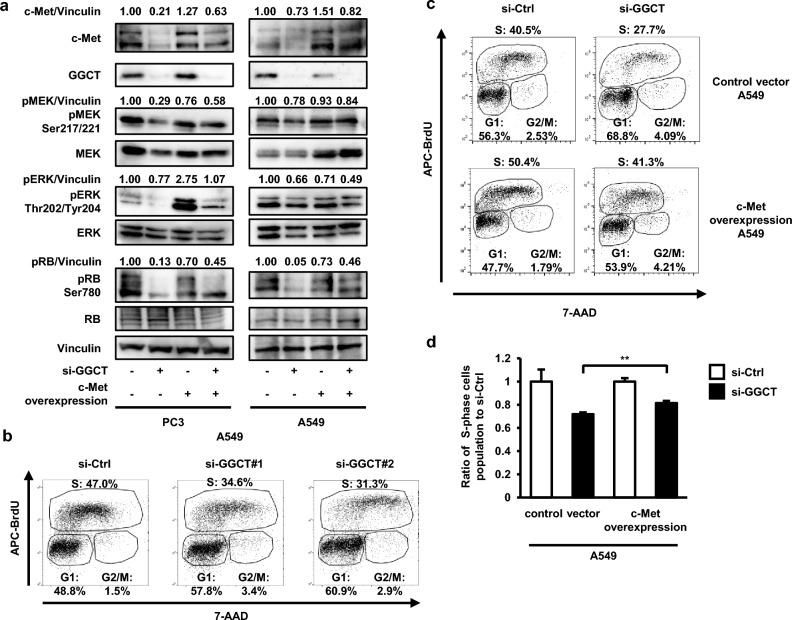
The knockdown of GGCT activates the RB protein by down-regulating c-Met. We established PC3 and A549 cells that stably overexpressed c-Met. (**a**) A Western blot analysis of c-Met, GGCT, pMEK Ser217/221, pERK Thr202/Tyr204, pRB Ser780, and their non-phosphorylated forms in control vector-transfected or c-Met-overexpressing-clone#1 of PC3 and A549 cells, treated with control siRNA or GGCT siRNA#2 for 72 h. Vinculin is shown as a loading control. All full-length blots are presented in Supplementary Fig. S6. (**b**) Fractions in the S phase of A549 cells at 48 h post-transfection with the indicated siRNAs were measured by a BrdU incorporation assay. (**c**) Fractions in the S phase of control vector-transfected or c-Met-overexpressing-clone#1 and A549 cells at 48 h post-transfection with control siRNA or GGCT siRNA#2 were measured by a BrdU incorporation assay. (**d**) The ratio of the population of S phase cells to si-Ctrl of each group (control vector or c-Met overexpression) of (**c**) are shown. A two-tailed Student’s *t*-test was used (N = 3 per group, **p < 0.01). The error bars represent the S. D.

We hypothesized that the knockdown of GGCT inhibited the MEK-ERK pathway and activated the RB protein by down-regulating c-Met. PC3 and A549 cells with the stable overexpression of c-Met were established, and after the depletion of GGCT, the phosphorylation levels of the MEK-ERK-RB pathway were analyzed by Western blotting. The results obtained showed that the inhibition of the MEK-ERK pathway induced by the knockdown of GGCT was restored in c-Met-overexpressing PC3 cells (Fig. 3a). In A549 cells, an apparent increase in pERK was not observed by the overexpression of c-Met, whereas pMEK was restored (Fig. 3a). Moreover, the dephosphorylation of RB induced by the knockdown of GGCT was inhibited by the overexpression of c-Met in PC3 and A549 cells (Fig. 3a, Supplementary Fig. S1). We analyzed the cell cycle using a BrdU incorporation assay. The knockdown of GGCT reduced the percentage of S phase cells (Fig. 3b, Supplementary Fig. S2). Although the growth-promoting effect of c-Met overexpression was evidently observed (from 40.5 to 50.4% in S phase, left panel of Fig. 3c), the growth-inhibitory effect of the knockdown of GGCT was significantly restored by c-Met overexpression (from 27.7 to 41.3% in S phase, right panel of Fig. 3c). In other words, the growth-inhibitory effect of the knockdown of GGCT was partially reduced to 81.4 ± 1.8% by overexpression of c-Met from 71.9 ± 1.5% in the control (Fig. 3d). No significant change in the ratio of the population of S phase cells was observed in PC3 cells (Supplementary Fig. S2). Therefore, it is likely that the decreased expression of c-Met is at least partially responsible for the growth-inhibitory effect of the knockdown of GGCT in A549 cells. Taken together, these results suggest that the knockdown of GGCT activated the RB protein, followed by cell cycle arrest through the down-regulation of c-Met in A549 cells.

### The knockdown of GGCT inhibits the phosphorylation and nuclear localization of STAT3

**Figure 4:**
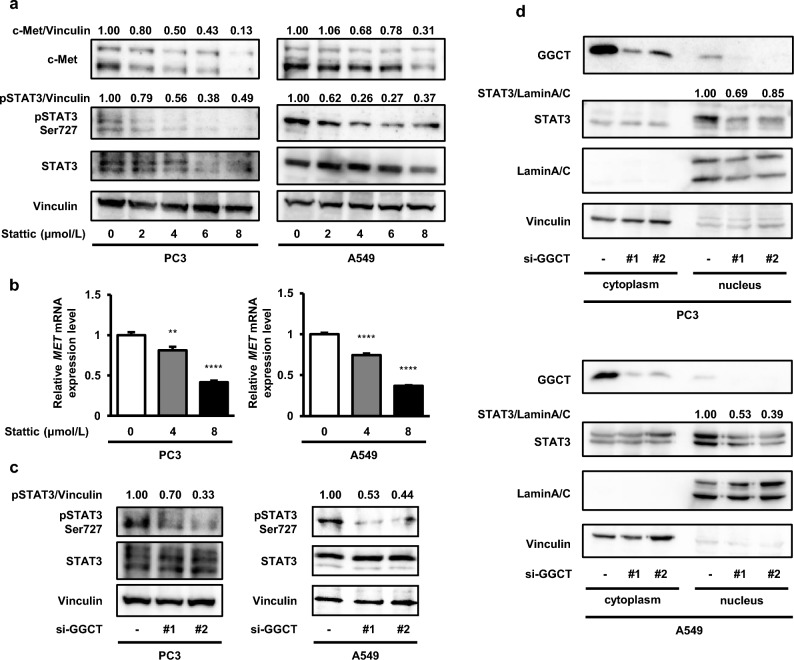
The knockdown of GGCT suppresses STAT3 phosphorylation and nuclear localization. (**a**) A Western blot analysis of c-Met, pSTAT3 Ser727, and STAT3 in PC3 and A549 cells treated with Stattic for 72 h (PC3) or 48 h (A549) at the indicated concentrations. Vinculin is shown as a loading control. All full-length blots are presented in Supplementary Fig. S7. (**b**) The expression of human-*MET* mRNA, as assessed by qRT-PCR, in PC3 and A549 cells at 72 or 48 h post-treatment with Stattic. A one-way ANOVA with Dunnett’s test was used (versus Stattic 0 μmol/L, N = 3 per group, **p < 0.01, ****p < 0.0001). The error bars represent the S. D. (**c**) A Western blot analysis of pSTAT3 Ser727, and STAT3 in PC3 and A549 cells treated with control siRNA or the indicated GGCT siRNAs for 72 h. Vinculin is shown as a loading control. All full-length blots are presented in Supplementary Fig. S7. (**d**) Proteins from PC3 and A549 cells treated with the indicated siRNAs for 72 h were fractionated into cytoplasmic and nuclear components. The protein expression of GGCT and STAT3 was analyzed by Western blotting. Lamin A/C and vinculin are shown as fractionation and loading controls. All full-length blots are presented in Supplementary Fig. S7.

The phosphorylation of STAT3 at Ser727 was shown to promote its translocation to the nucleus^18^, and nuclear STAT3 bound to the promoter region of the *MET* gene^19^. A treatment with Stattic, an inhibitor of STAT3, decreased c-Met protein and mRNA expression in PC3 and A549 cells (Fig. 4a,b), suggesting that STAT3 up-regulated c-Met expression in these cell lines. Since the knockdown of GGCT down-regulated c-Met protein and mRNA expression, we hypothesized that GGCT may promote the function of STAT3 as a transcription factor. The phosphorylation of STAT3 at Ser727 was inhibited in GGCT-depleted PC3 and A549 cells (Fig. 4c), suggesting that the knockdown of GGCT suppressed the function of STAT3 by inhibiting its phosphorylation. This result is supported by the knockdown of GGCT decreasing the nuclear expression of STAT3 in PC3 and A549 cells (Fig. 4d).

### The knockdown of GGCT down-regulates c-Met expression and activates RB through AMPK activation in PC3 cells

**Figure 5:**
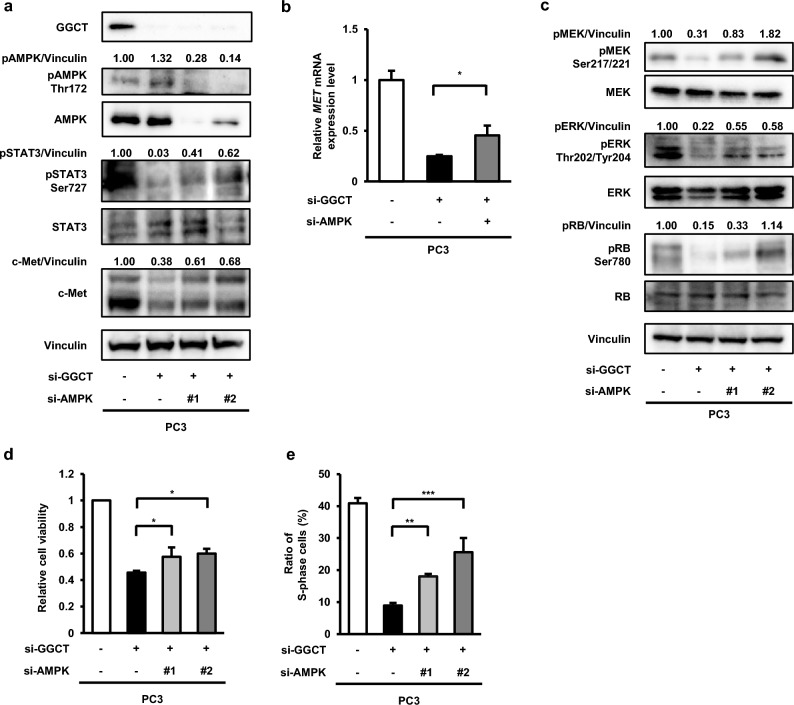
The knockdown of GGCT down-regulates pSTAT3 and c-Met and activates the RB protein via AMPK. (**a**) A Western blot analysis of GGCT, c-Met, pAMPK Thr172, pSTAT3 Ser727, and their non-phosphorylated forms in PC3 cells treated with control siRNA, GGCT siRNA#2, and/or the indicated AMPK siRNAs for 72 h. Vinculin is shown as a loading control. All full-length blots are presented in Supplementary Fig. S8. (**b**) The expression of human-*MET* mRNA, as assessed by qRT-PCR, in PC3 cells at 72 h post-transfection with control siRNA, GGCT siRNA#2, and/or AMPKα siRNA#2. A two-tailed Student’s *t*-test was used (N = 3 per group, *p < 0.05). The error bars represent the S. D. (**c**) A Western blot analysis of pMEK Ser217/221, pERK Thr202/Tyr204, pRB Ser780, and their non-phosphorylated forms in PC3 cells treated with control siRNA, GGCT siRNA#2, and/or the indicated AMPK siRNAs for 72 h. Vinculin is shown as a loading control. All full-length blots are presented in Supplementary Fig. S8. (**d**) The viable cell numbers of PC3 cells treated with control siRNA, GGCT siRNA#2, and/or the indicated AMPK siRNAs for 72 h were analyzed by the trypan blue dye exclusion test. A one-way ANOVA with Dunnett’s test was used (N = 3 per group, *p < 0.05). The error bars represent the S. D. (**e**) Fractions in the S phase of PC3 cells at 48 h post-transfection with indicated siRNAs were measured by a BrdU incorporation assay. A one-way ANOVA with Dunnett’s test was used (N = 3 per group, **p < 0.01, ***p < 0.001). The error bars represent the S. D.

We previously reported that the knockdown of GGCT activated AMPK^13,20^, and that the concurrent knockdown of AMPK and GGCT recovered the growth inhibition of GGCT-depleted PC3 cells^21^. We herein demonstrated that the simultaneous knockdown of AMPK and GGCT restored the dephosphorylation of STAT3 (Fig. 5a). Consistent with this result, the knockdown of AMPK significantly restored c-Met protein and mRNA expression (Fig. 5a,b). Moreover, the inhibition of the MEK-ERK pathway in GGCT-depleted PC3 cells was restored by the knockdown of AMPK (Fig. 5c). The activation of the RB protein by the knockdown of GGCT was also attenuated by the simultaneous knockdown of AMPK (Fig. 5c). Consistent with the previous report^21^, the knockdown of AMPK recovered the number of viable cells and S phase cells decreased by depleting GGCT (Fig. 5d,e). These results indicate that the knockdown of GGCT in PC3 cells inactivated STAT3, down-regulated c-Met at the transcriptional level, and inhibited the MEK-ERK pathway followed by the activation of the RB protein in an AMPK-dependent manner. On the other hand, the knockdown of AMPK did not restore the down-regulation of c-Met in GGCT-depleted A549 cells (Supplementary Fig. S3).

### The inhibition of GGCT suppresses the expression of c-Met and pRB in a mouse xenograft model of PC3 cells

**Figure 6:**
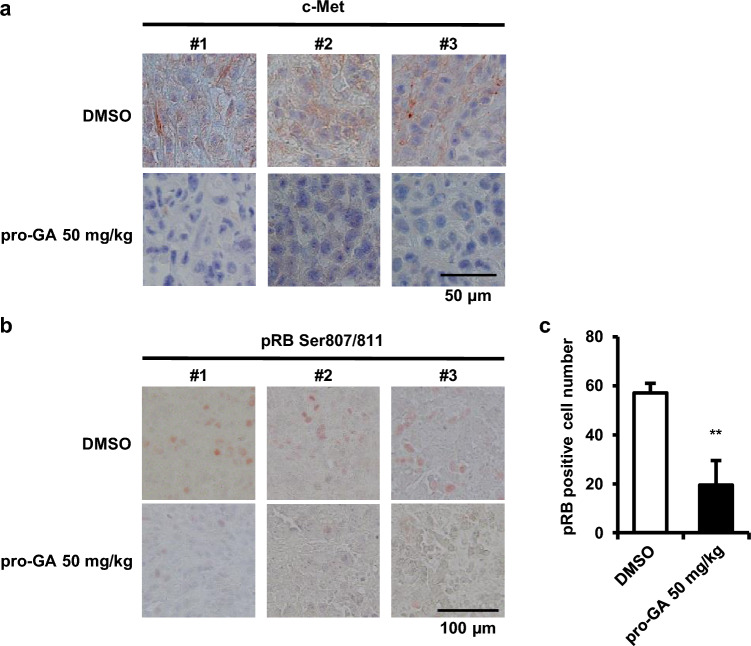
A treatment with pro-GA, a GGCT inhibitor, down-regulates the expression of c-Met and pRB in a mouse xenograft tumor model. Mice were treated with pro-GA or DMSO twice a week for 5 weeks. (**a**) Tumor sections were analyzed by immunohistochemistry with antibodies against c-Met (blue: nuclear; brown: c-Met). Magnification 400-fold. Scale bar 50 μm. (**b**) Tumor sections were analyzed by immunohistochemistry with antibodies against pRB Ser807/811 (brown). Magnification 200-fold. Scale bar 100 μm. (**c**) The number of pRB-positive cells in (**b**). A two-tailed Student’s t-test was used (N = 3 per group, **p < 0.01). The error bars represent the S. D.

Pro-GA, our original inhibitor targeting GGCT, significantly inhibited tumor growth in a mouse xenograft model of PC3 cells^22^. We performed immunohistochemistry to investigate whether the inhibition of GGCT affected the expression of c-Met and pRB in xenograft tumors. The intraperitoneal administration of pro-GA (50 mg/kg) to mice suppressed c-Met protein expression in tumors, indicating that GGCT regulated c-Met expression not only in vitro, but also in vivo (Fig. 6a). RB has multiple phosphorylation sites, and phosphorylation at both Ser780 and Ser807/811 causes RB inactivation^23,24^. The tumor tissues derived from xenograft mice treated with pro-GA showed lower expression of pRB Ser807/811 compared to control, suggesting that the inhibition of GGCT activates the RB protein also in vivo (Fig. 6b,c).

## Discussion

The activation of a number of oncogenes inactivates RB at the protein level, and clinically available inhibitors of each oncogene activate the RB protein, which is recognized as one of the most essential therapeutic and preventive molecules^12^. We herein demonstrated that the knockdown of GGCT activated the function of RB by inactivating the MEK-ERK pathway in multiple human cancer cell lines (Fig. 1b,c).

He et al. recently reported that KRAS-driven lung tumorigenesis was inhibited in a GGCT-knockout mouse model without affecting normal development and tissue function^25^. Therefore, the high expression of GGCT may promote tumorigenesis by inactivating RB, and GGCT is considered to be a promising target of cancer therapy and prevention. The development of GGCT inhibitors has grown in recent years. We developed the first GGCT inhibitor, pro-GA, which exerted significant antitumor effects without severe systemic adverse effects in a mouse xenograft model of PC3 cells^22^. Ii et al. additionally identified U83836E as a novel GGCT inhibitor using LISA-101, a fluorescent probe specific for GGCT^26,27^, and U83836E suppressed tumor growth in a mouse xenograft model of MCF7 human breast cancer cells without a reduction in body weight^26^.

In the present study, the knockdown of GGCT down-regulated c-Met, while its overexpression in NIH3T3 cells up-regulated c-Met, indicating that c-Met is a novel downstream target of GGCT (Fig. 2). Consistently, the overexpression of c-Met attenuated the phenomena induced by the knockdown of GGCT, such as the inhibition of the MEK-ERK pathway and the activation of the RB protein (Fig. 3a). In addition, the decrease of the population of S phase cells in GGCT-depleted A549 cells was significantly recovered by c-Met overexpression, indicating that the cell cycle arrest was induced by the knockdown of GGCT, at least in part, through the downregulation of c-Met (Fig. 3c,d). Furthermore, the treatment with pro-GA, a GGCT inhibitor, reduced c-Met protein expression and activated the RB protein in tumor-bearing mice of PC3 cells (Fig. 6). c-Met belongs to the family of tyrosine kinase receptors and is activated by its native ligand HGF^16,17^. The mutation, gene amplification, and overexpression of c-Met frequently occur in various malignant tumors^16,17^. Therefore, the antitumor effects of the inhibition of GGCT are considered to be dependent on the down-regulation of c-Met.

The overexpression of c-Met recovered growth inhibition with cell cycle arrest in GGCT-depleted A549 cells (Fig. 3d). In addition, in GGCT-depleted PC3 cells, the similar effect of c-Met overexpression on the cell cycle was observed, but not significant (Supplementary Fig. S2). This is probably because the inhibition of cancer cell growth induced by the knockdown of GGCT depends not only on the down-regulation of c-Met, but also on other mechanisms in PC3 cells. We previously reported that the knockdown of GGCT up-regulated the CDK inhibitors, p21^Waf1/Cip1^ and p16^INK4A^, causing cell cycle arrest^28^. Particularly, in PC3 and glioblastoma A172 cells, we additionally showed that the up-regulation of p21^Waf1/Cip1^ was induced by the activation of the transcription factor forkhead box O3a (FOXO3a)^21^. In this study, the knockdown of FOXO3a significantly rescued the growth inhibition induced by the knockdown of GGCT^21^, suggesting that the depletion of GGCT suppresses the growth of PC3 cells through multiple molecular mechanisms, and that the alternative pathways other than c-Met may dominantly contribute to the growth-inhibitory effect of the knockdown of GGCT.

In A549 cells, c-Met overexpression did not markedly rescue the dephosphorylation of ERK, whereas the activation of the RB protein and the cell cycle arrest were significantly attenuated (Fig. 3a). These results suggest that the down-regulation of c-Met in GGCT-depleted A549 cells activated the RB protein in a MAPK-independent manner. The PI3K-AKT signaling pathway is also activated by c-Met^17^. Activated AKT has been reported to directly phosphorylate the CDK inhibitors p21^Waf1/Cip1^^29^ and p27^Kip1^^30^, and suppress their functions, leading to the inactivation of the RB protein. On the other hand, Zhang et al*.* reported that GGCT interacts with mitochondrial ribosomal protein L9 (MRPL9) and their simultaneous knockdown suppresses the phosphorylation level of ERK in papillary thyroid cancer cells^31^. Thus, knockdown of GGCT may also inhibit ERK phosphorylation by disrupting its interaction with MRPL9, limiting the restorative effect of c-Met overexpression on the ERK dephosphorylation.

Zhu et al. reported that STAT3 bound to the promoter region of the *MET* gene and activated its transcription in breast cancer cells with multi-drug resistance^19^. The function of STAT3 as a transcription factor is regulated by its phosphorylation and dimerization, followed by nuclear translocation^32^. The phosphorylation of STAT3 at Ser727 is necessary for the maximal activation of STAT3 as a transcription factor^32^, which is regulated by multiple kinases, including mTORC1^33^. Phosphorylation at Ser727 was previously shown to promote nuclear localization with the transcriptional activity of STAT3 in chronic lymphoid leukemia cells^18^. We herein demonstrated that the knockdown of GGCT decreased phosphorylation at Ser727 and the nuclear expression of STAT3 in PC3 and A549 cell lines (Fig. 4c,d). Therefore, the knockdown of GGCT may down-regulate c-Met expression by inactivating STAT3 as a transcription factor. This concept is supported by c-Met protein and mRNA expression being down-regulated by the treatment with Stattic, a STAT3 inhibitor, in PC3 and A549 cells (Fig. 4a,b).

**Figure 7:**
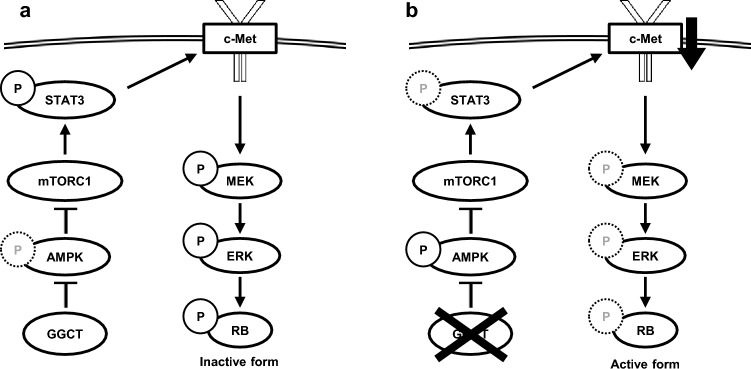
A proposed graphical summary of the signaling cascades involved in down-regulation of c-Met upon the knockdown of GGCT in PC3 cells. (**a**) In many tumor cells, highly expressed GGCT inactivates RB via c-Met and proposed signaling cascades. (**b**) Depletion or inhibition of GGCT results in down-regulation of c-Met via the AMPK-STAT3 pathway, followed by RB activation.

We previously reported that the knockdown of GGCT inhibited mTORC1 by activating AMPK^13,20^. In the present study, the knockdown of AMPK restored the dephosphorylation of STAT3 at Ser727, which has been phosphorylated by mTORC1^34^, and the down-regulation of c-Met in GGCT-depleted PC3 cells (Fig. 5a). Consistent with this result, the inhibition of the MEK-ERK pathway and the activation of the RB protein by the knockdown of GGCT were also reversed by the knockdown of AMPK in PC3 cells (Fig. 5c). The knockdown of AMPK significantly attenuated the down-regulation of c-Met mRNA in GGCT-depleted PC3 cells (Fig. 5b), which supports the hypothesis that GGCT up-regulated c-Met expression at the transcriptional level via AMPK-STAT3. Therefore, the knockdown of GGCT is considered to inhibit the phosphorylation of STAT3, up-regulation of c-Met, and activation of the MEK-ERK pathway in an AMPK-mTORC1-dependent manner at least in PC3 cells (Fig. 7). In contrast, the knockdown of AMPK did not restore the down-regulation of c-Met in GGCT-depleted A549 cells, suggesting that the decrease of c-Met expression caused by the knockdown of GGCT is regulated by other mechanisms in A549 cells (Supplementary Fig. S3). The mechanism by which the knockdown of GGCT down-regulates c-Met may differ depending on cell types.

In summary, we identified c-Met as a novel target of GGCT and showed that it is a key molecule mediating the inhibition of GGCT to the activation of the RB protein through the GGCT-AMPK-mTORC1-STAT3-c-Met-MEK-ERK-RB axis (Fig. 7). The present results revealed one of the essential mechanisms by which the high expression of GGCT in a number of malignancies causes carcinogenesis, and highlight the potential for cancer therapeutic and preventive strategies targeting GGCT.

## Methods

### Cell culture

Prostate PC3 and renal A498 cancer cell lines were obtained from the NCI-60 panel (National Cancer Institute, Bethesda, MD, USA). Bladder J82 and lung A549 cancer cell lines were purchased from the American Type Culture Collection (ATCC) (Rockville, MD, USA). NIH3T3 mouse embryonic fibroblasts were purchased from ATCC, and GGCT was overexpressed using the pCX4bsr retroviral vector as previously described^2^. PC3, J82, and NIH3T3 cells were cultured in Dulbecco’s modified Eagle’s medium (#043-30085, Wako Pure Chemical Industries, Osaka, Japan), A498 cells in Eagle’s minimum essential medium (#055-08975, Wako), and A549 cells in RPMI-1640 (#05918, Nissui, Tokyo, Japan) supplemented with 300 μg/mL _L_-glutamine and 1 mmol/L sodium pyruvate. All growth media were supplemented with 10% FBS (#104037, Gibco, Waltham, MA, USA), 50 units/mL penicillin, and 100 μg/mL streptomycin. All cells were maintained at 37 °C in a 5% CO_2_ atmosphere.

### siRNA transfection

All High-purity Custom siRNAs were purchased from Qiagen (#1027423, Hilden, Germany). AllStars Neg. Control siRNA (#1027281, Qiagen) was used as the non-targeting control. siRNAs were transiently transfected with Lipofectamine RNAi MAX (#13778150, Thermo Fisher Scientific, Waltham, MA, USA). All siRNAs were transfected at a concentration of 16.7 nmol/L. In experiments in which GGCT and AMPK were simultaneously knocked down, 33.4 nmol/L non-targeting siRNA was transfected as the control. siRNAs targeting the following sequences were used: GGCT#1, 5′-CCTGTCGAAGTTATCTGATGA-3′; GGCT#2, 5′-AATGACTATACAGGAAAGGTC-3′; AMPKα#1, 5′-AATGCCTACCATCTCATAATA-3′; AMPKα#2, 5′-AACCCATATTATTTGCGTGTA-3′. Unless otherwise noted, GGCT siRNA #2 and/or AMPKα siRNA #2 were used.

## Cell viability assay

PC3, J82, A498, and A549 cells were seeded on 96-well plates at 500 cells per well, incubated for 1 day, and transfected with the indicated siRNAs. After a 120-h incubation, cell viability assays were performed with Cell Counting Kit-8 (CCK-8) (#341-08001, DOJINDO, Kumamoto, Japan).

### Antibodies and reagents

The following rabbit monoclonal antibodies were purchased from Cell Signaling Technology (CST) (Danvers, MA, USA): pMEK1/2 Ser217/221 (1:1000, #9154); MEK1/2 (1:1000; #9122); pERK1/2 Thr202/Tyr204 (1:1000, #4370); ERK1/2 (1:1000, #9102); pRB Ser780 (1:1000, #9307); pRB Ser807/811 (1:200, #9308); STAT3 (1:1000, #4904); pAMPKα Thr172 (1:1000, #2535); AMPKα (1:1000, #5832), and Lamin A/C (1:1000, #2032). An anti-GGCT rabbit monoclonal antibody was purchased from Abcam (1:1000, #ab198503, Cambridge, UK). Mouse monoclonal antibodies against the following proteins were used: RB (1:2000, #9309, CST); vinculin (1:2000, #66305, ProteinTech, Rosemont, IL, USA); c-Met (1:1000, #3127, CST); tubulin (1:1000, #CP06, Calbiochem, Burlington, MA, USA); pSTAT3 Ser727 (1:2000, #9136, CST). Horseradish peroxidase (HRP)-linked donkey anti-rabbit IgG and HRP-linked sheep anti-mouse IgG were purchased from Cytiva (1:2000, #NA934 and #NA931; Tokyo, Japan). Stattic, an inhibitor of STAT3, was purchased from Abcam (#19983-44-9). Pro-GA, an inhibitor of GGCT, was purchased from Funakoshi (#FDV-0019, Tokyo, Japan).

### Western blot analysis

Cells were washed with PBS and lysed with lysis buffer (150 mmol/L NaCl, 50 mmol/L tris-base, 1% NP-40, 0.5% sodium deoxycholate, and 0.1% SDS) supplemented with a protease inhibitor cocktail mix (Nacalai Tesque, Kyoto, Japan) and phosphatase inhibitor cocktail mix (Nacalai Tesque). 5 × sample buffer (125 mmol/L tris-based pH 6.8, 20% glycerol, 10% 2-mercaptoethanol, 4% SDS, and 0.04% bromophenol blue) was added to the lysate and the mixtures were heated at 95 °C for 5 min. An equal amount of the protein extract was separated by SDS-PAGE and transferred to polyvinylidene difluoride membranes (Millipore, Billerica, MA, USA). Membranes were blocked with 5% fat-free dry milk or 5% bovine serum albumin in TBS with 0.1% Tween-20 (TBST) and then incubated with the indicated primary and secondary antibodies in Signal Enhancer HIKARI (#02270-81, Nacalai Tesque). To detect pSTAT3, membranes were blocked with 7% fat-free dry milk in TBST. Proteins were visualized using the Immobilon Western Chemiluminescent HRP Substrate (#WBKLS0500, Millipore) or Chemi-Lumi One L (#07880, Nacalai Tesque). Chemiluminescence was detected using the ChemiDoc Touch imaging system (Bio-Rad Laboratories, Hercules, CA, USA). Protein bands were quantified with the Image Lab Software (Bio-Rad).

### Reverse transcription-quantitative PCR (RT-qPCR) analysis

Total RNA was extracted from cells lysed with Sepasol-RNA I Super G (#09379-84, Nacalai Tesque) and purified according to the manufacturer’s protocol. cDNA was synthesized using the ReverTra Ace qPCR RT Master Mix (#FSQ-201, TOYOBO, Osaka, Japan). Synthesized cDNA was analyzed by TaqMan Gene Expression Assays (Thermo Fisher Scientific) and THUNDERBIRD Probe qPCR Mix (#QPS-101, TOYOBO) using the QuantStudio 3 System (Thermo Fisher Scientific). PCR conditions were as follows: cycle 1: 50 °C for 2 min; cycle 2: 95 °C for 10 min; cycle 3 (× 40): 95 °C for 15 s and 60 °C for 1 min. Gene expression levels were normalized against human-*B2M* or mouse-*Actb*, a housekeeping gene used as an internal control. The following TaqMan probes were purchased from Thermo Fisher Scientific: human-*MET* (Hs01565584_m1), human-*B2M* (Hs00984230_m1), mouse-*Met* (Mm01156972_m1), and mouse-*Actb* (Mm02619580_g1).

### Microarray analysis

Microarray analyses were performed as previously described^13^. Briefly, total RNA samples were purified using TRIzol (#15596026, Thermo Fisher Scientific) and the RNeasy Mini Kit (#74104, Qiagen) from PC3 cells treated with siRNA targeting GGCT#2 or non-targeting siRNA, and NIH3T3 cells overexpressing GGCT or the control. Expression profiling was performed using SurePrint G3 Human GE 8 × 60 K v3 and Whole Mouse Genome SurePrint G3 Mouse Gene Expression 8 × 60 K v2 (Agilent Technologies, Santa Clara, CA, USA). Data were analyzed using GeneSpring software (version 14.9.1; Silicon Genetics, Redwood city, CA, USA). Well-established genes activating growth factor signaling were extracted from a gene set that was significantly down-regulated by the knockdown of GGCT and on that was significantly up-regulated by its overexpression, and a Venn diagram of the overlap between these gene sets was drawn.

### Preparation of c-Met-overexpressing cells

The pCMV3-C-FLAG-c-Met vector and its empty vector were obtained from Sino Biological (HG10692-CF and CV012, Beijing, China). PC3 and A549 cells were seeded on 6-well plates at 2.5 × 10^5^ cells per well, incubated for 1 day, and transfected with 2.5 mg of the pCMV3-C-FLAG-c-Met vector or its empty vector using Lipofectamine 3000 (#L3000015, Thermo Fisher Scientific). At 3 days post-transfection, cells were incubated in growth medium containing 200 μg/mL Hygromycin B Gold (#ant-hg-1, InvivoGen, San Diego, CA, USA) for 2 weeks to select vector-transfected cells. Two monoclonal cell lines (c-Met overexpressing-clone#1 and clone#2) were obtained using a limiting dilution-cloning method; vector-transfected cells were seeded on a 96-well plate at 0.5 cells per well and incubated for a few weeks. Unless otherwise noted, colne#1 was used.

### Trypan blue dye exclusion test

PC3 cells were seeded on 6-well plates at 5 × 10^4^ cells per well, incubated for 1 day, and transfected with the indicated siRNAs. After a 72-h incubation, a trypan blue dye exclusion test was performed using 0.4% trypan blue solution (#EBT-001, NanoEntek, Gyeonggi-do, Korea) and the EVE Automated Cell Counter (NanoEntek).

### Bromodeoxyuridine (BrdU) incorporation assay

PC3 and A549 cells were seeded on 6-cm dishes at 1.2 × 10^5^ cells per dish, incubated for 1 day, and transfected with the indicated siRNAs for 48 h. The percentage of PC3 and A549 cells in the S phase was assessed using an FITC or APC BrdU flow kit (#559619 or #552598, BD Biosciences, Franklin Lakes, NJ, USA) according to the manufacturer’s protocol. Briefly, after 10 μmol/L BrdU-pulse for 3 h, cells were collected and washed. After fixing cells and membrane permeabilization, cells were treated with 300 μg/mL DNase at 37 °C for 1 h. A FITC- or APC-conjugated anti-BrdU antibody was added to cells. Nuclear staining was performed using 7-AAD. The amount of incorporated BrdU was analyzed using the BD Accuri C6 Plus flow cytometer (BD Biosciences). At least 10,000 cells per sample were counted, and data were analyzed using FlowJo software (BD Biosciences).

### Fractionation of nuclear/cytoplasmic proteins

PC3 and A549 cells were seeded on 10-cm dishes at 3 × 10^5^ cells per dish, incubated for 1 day, and transfected with the indicated siRNAs. After a 72-h incubation, cellular proteins were fractionated into cytoplasmic and nuclear fractions using the Nuclear Extract Kit (#40010, Active Motif, Carlsbad, CA, USA) according to the manufacturer’s protocol. Briefly, cells were collected and lysed in 500 μL of hypotonic buffer. After a 15-min incubation on ice, 25 μL of detergent was added and lysates were stirred with a vortex mixer for 10 s. After centrifugation at 14,000×*g* at 4 °C for 1 min, the supernatants were collected (cytoplasmic fraction). Nuclear pellets were resuspended with complete lysis buffer and shaken at 1200 rpm at 4 °C for 30 min. After centrifugation at 14,000×*g* at 4 °C for 10 min, the supernatants were collected (nuclear fraction).

### Xenograft model study

All experiments were performed in accordance with the ARRIVE guidelines 2.0 and the Guide for the Care and Use of Laboratory Animals (National Research Council, 8th edition, 2011), and were performed under the approval of the Institutional Ethical Committee for Animal Experiments of Kyoto Pharmaceutical University (Approval number: CLON-19-001). The procedure was previously described^22^. Briefly, male CB-17 severe combined immunodeficient mice (5 weeks old, n = 3 per group) were purchased from Japan SLC (Shizuoka, Japan). Mice were subcutaneously inoculated with 2.5 × 10^6^ PC3 cells in PBS containing Matrigel (1:1, Corning, NY, USA) under isoflurane inhalation anesthesia, and then randomized immediately. Mice were intraperitoneally administered pro-GA (50 mg/kg) or DMSO in 50 μL of olive oil twice a week for 5 weeks. When excising tumor tissues, euthanasia was given to mice by cervical dislocation under isoflurane inhalation anesthesia.

### Immunohistochemistry

Tumor tissue derived from mouse xenograft models of PC3 cells was fixed with phosphate-buffered 10% formalin, embedded in paraffin, cut into 4-μm-thick slices, and then deparaffinized using xylene. After heating in an antigen retrieval reagent (10 mmol/L sodium citrate solution, pH 6.0) for 30 min and standing at room temperature for 20 min, sections were treated with 3% hydrogen peroxide in TBS at room temperature for 10 min to inhibit endogenous peroxidase. After being rinsed with TBS, sections were blocked with 10% normal goat serum at room temperature for 1 h and then incubated at 4 °C for 48 h with a mouse monoclonal antibody against c-Met (1:400). For pRB Ser807/811 detection, the sections were incubated at 4 °C for overnight with rabbit polyclonal antibody against pRB Ser807/811 (1:200). Sections were washed with TBS and treated with EnVision + system HRP-labeled polymer anti-mouse (#K4001, DAKO, Carpinteria, CA, USA) or anti-rabbit (#K4003, DAKO), and incubated at room temperature for 30 min. The bound antibodies were visualized with AEC + high-sensitivity substrate chromogen (#K3469, DAKO) and counterstained with Mayer’s hematoxylin. The number of pRB-positive cells was counted in at least 3 fields for each sample, and the average was calculated.

### Statistical analysis

All data were obtained from at least three independent experiments and expressed as the mean ± S.D. Statistical analyses were performed using a two-tailed Student’s *t*-test or one-way ANOVA with Dunnett’s multiple comparison test by Graph pad prism 9 software (Dotmatics, San Diego, CA, USA). Regarding the microarray expression profile data of PC3 cells (control versus GGCT siRNA) and NIH3T3 cells (control versus GGCT overexpression), Welch’s unpaired *t*-test was performed to calculate *p* values using GeneSpring 14.9 software. *p* values < 0.05 were considered to be significant.

## Supplementary Information

Supplementary Figures.

## Data Availability

The datasets analyzed during the current study are available in the Gene Expression Omnibus database (GSE154584, PC3, https://www.ncbi.nlm.nih.gov/geo/query/acc.cgi?acc=GSE154584; GSE154592, NIH3T3, https://www.ncbi.nlm.nih.gov/geo/query/acc.cgi?acc=GSE154592). We will provide all data and protocols described in the manuscript upon reasonable request.
