# Supplementary material for: Identification of c-Met as a novel target of γ-glutamylcyclotransferase
Source: Sci Rep. 2023 Jul 24;13:11922. doi: 10.1038/s41598-023-39093-7 (PMC10366151; doi:10.1038/s41598-023-39093-7)
Supplement: Supplementary file 1 — Supplementary Figures. [file 41598_2023_39093_MOESM1_ESM.pdf]

Supplementary Figures

"Identification of c-Met as a novel target of γ-glutamylcyclotransferase"

Yumiko Saito[1,2†], Keiko Taniguchi[1*†,] Hiromi Ii[3], Mano Horinaka[1], Susumu Kageyama[4], Susumu Nakata[3], Osamu Ukimura[2], Toshiyuki Sakai[1].

[1]Department of Drug Discovery Medicine, Kyoto Prefectural University of Medicine, Kyoto, Japan.
[2]Department of Urology, Kyoto Prefectural University of Medicine, Kyoto, Japan.
[3]Department of Clinical Oncology, Kyoto Pharmaceutical University, Kyoto, Japan.
[4]Department of Urology, Shiga University of Medical Science, Shiga, Japan.

[*]Corresponding author
[†]These authors contributed equally to this work.

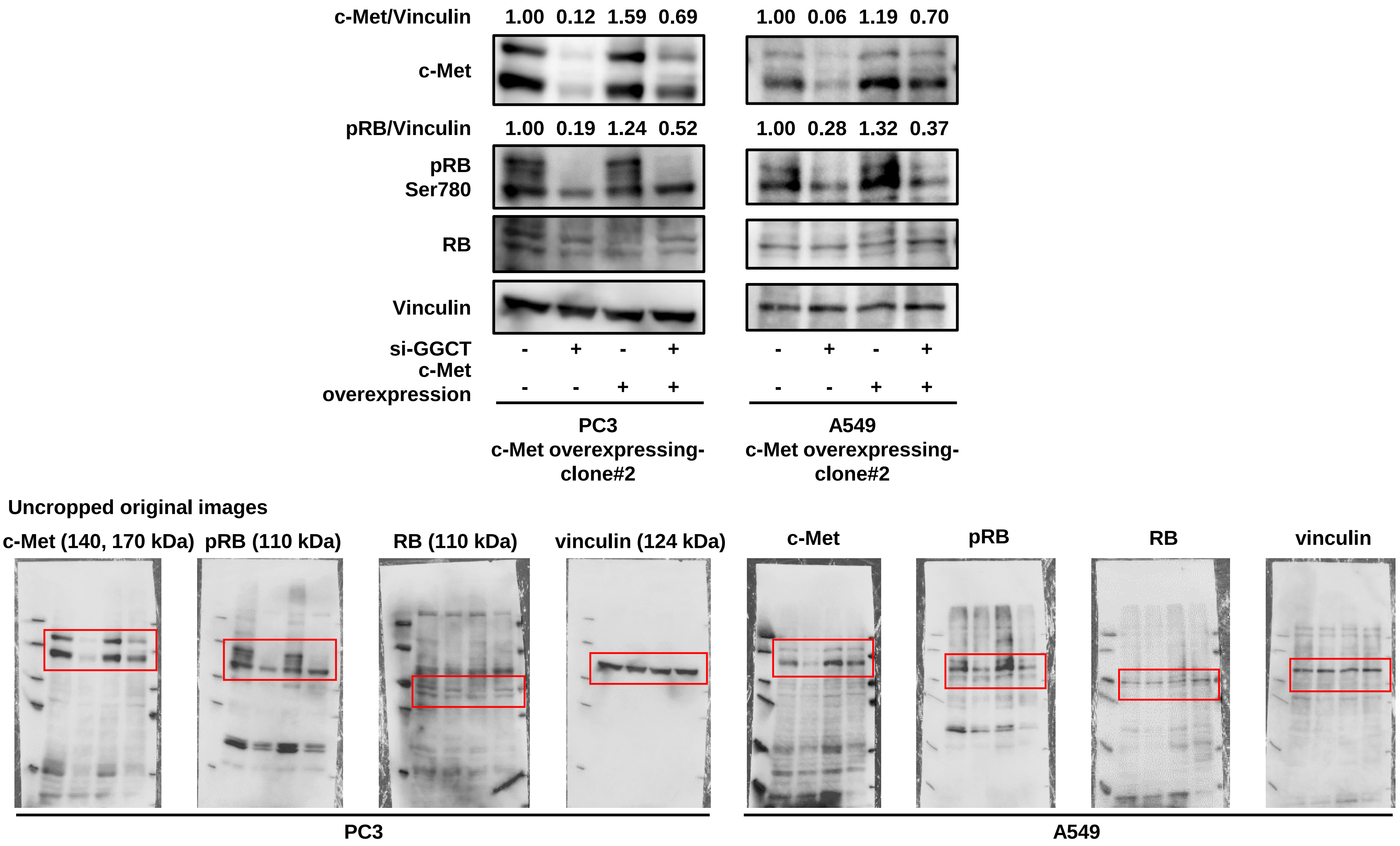

Supplementary Figure S1. c-Met overexpression attenuated RB activation and cell growth inhibition in another clone of PC3 and A549 cells. Western blot analysis of c-Met, pRB Ser780, and RB in control vector-transfected or c-Met-overexpressing clone#2 of PC3 and A549 cells, treated with control siRNA or GGCT siRNA#2 for 72 h. Vinculin is shown as a loading control.

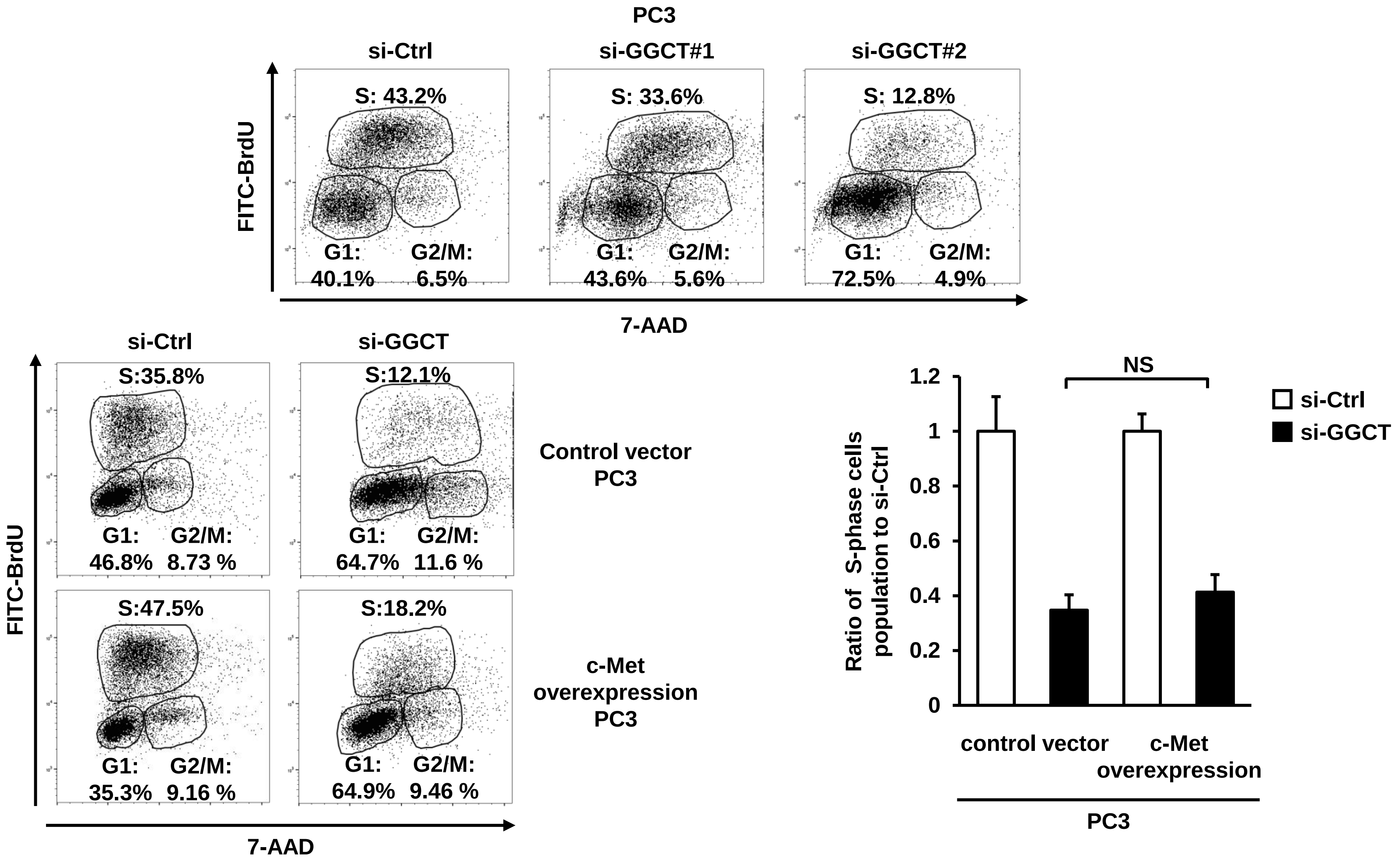

Supplementary Figure S2. Upper panel: Fractions in the S-phase of PC3 cells at 48 h post-transfection with the indicated siRNAs were measured by a BrdU incorporation assay. Lower panel: Fractions in the S-phase of control vector-transfected or c-Met-overexpressing-clone#1 and PC3 cells at 48 h post-transfection with control siRNA or GGCT siRNA#2 were measured by a BrdU incorporation assay. The ratio of the population of S-phase cells to si-Ctrl of each group (control vector or c-Met overexpression) are shown. A two-tailed Student's t-test was used (N=3 per group; NS, not significant). The error bars represent the S. D.

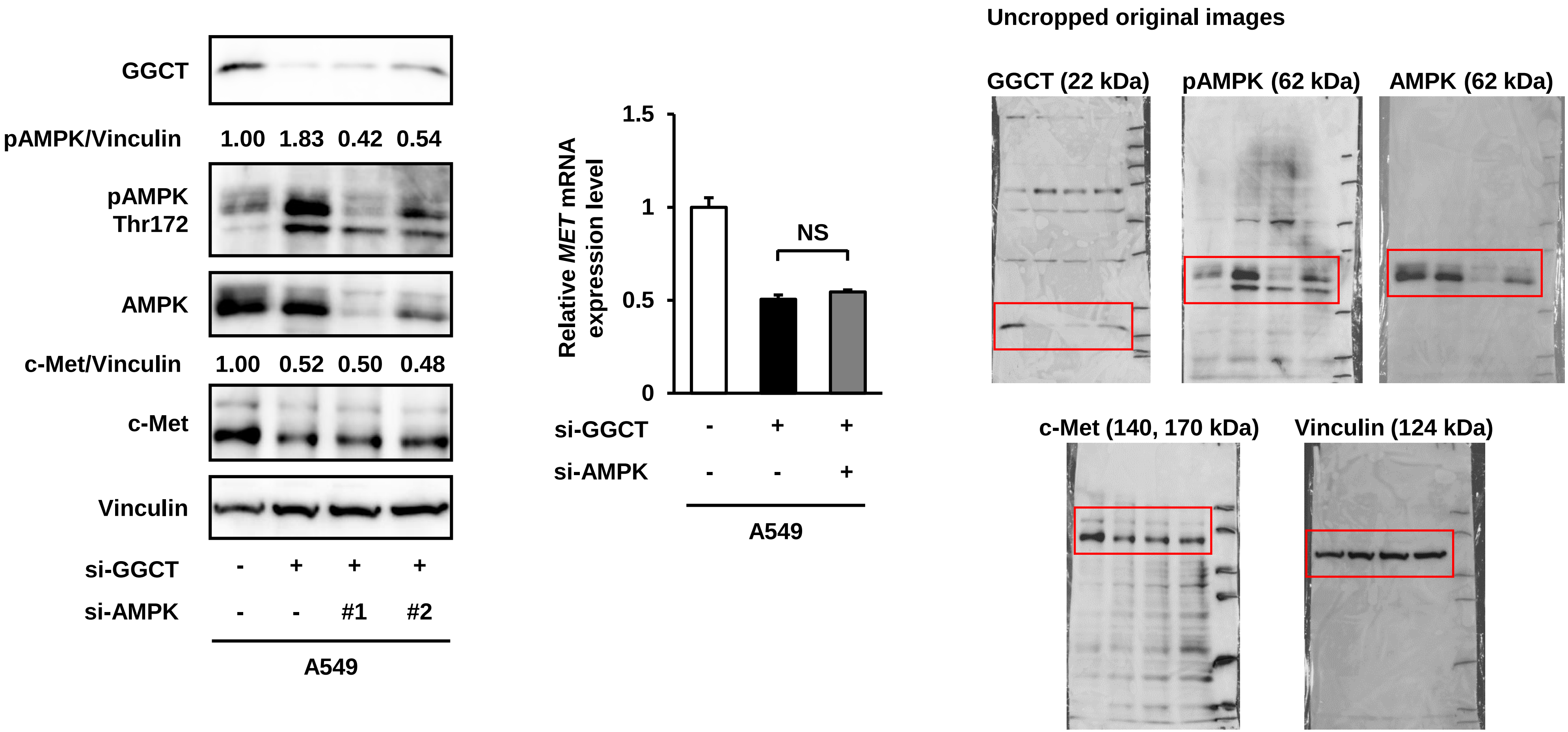

Supplementary Figure S3. The knockdown of AMPK did not restore the down-regulation of c-Met in GGCT-depleted A549 cells. Western blot analysis of GGCT, pAMPK Thr172, AMPK and c-Met in A549 cells treated with control siRNA, GGCT siRNA#2, and/or the indicated AMPK siRNAs for 72 h. Vinculin is shown as a loading control. The expression of human-*MET* mRNA, as assessed by qRT-PCR, in A549 cells at 72 h post-transfection with control siRNA, GGCT siRNA#2, and/or AMPK#2 siRNAs. A two-tailed Student's t-test was used (N=3 per group; NS, not significant). The error bars represent the S. D.

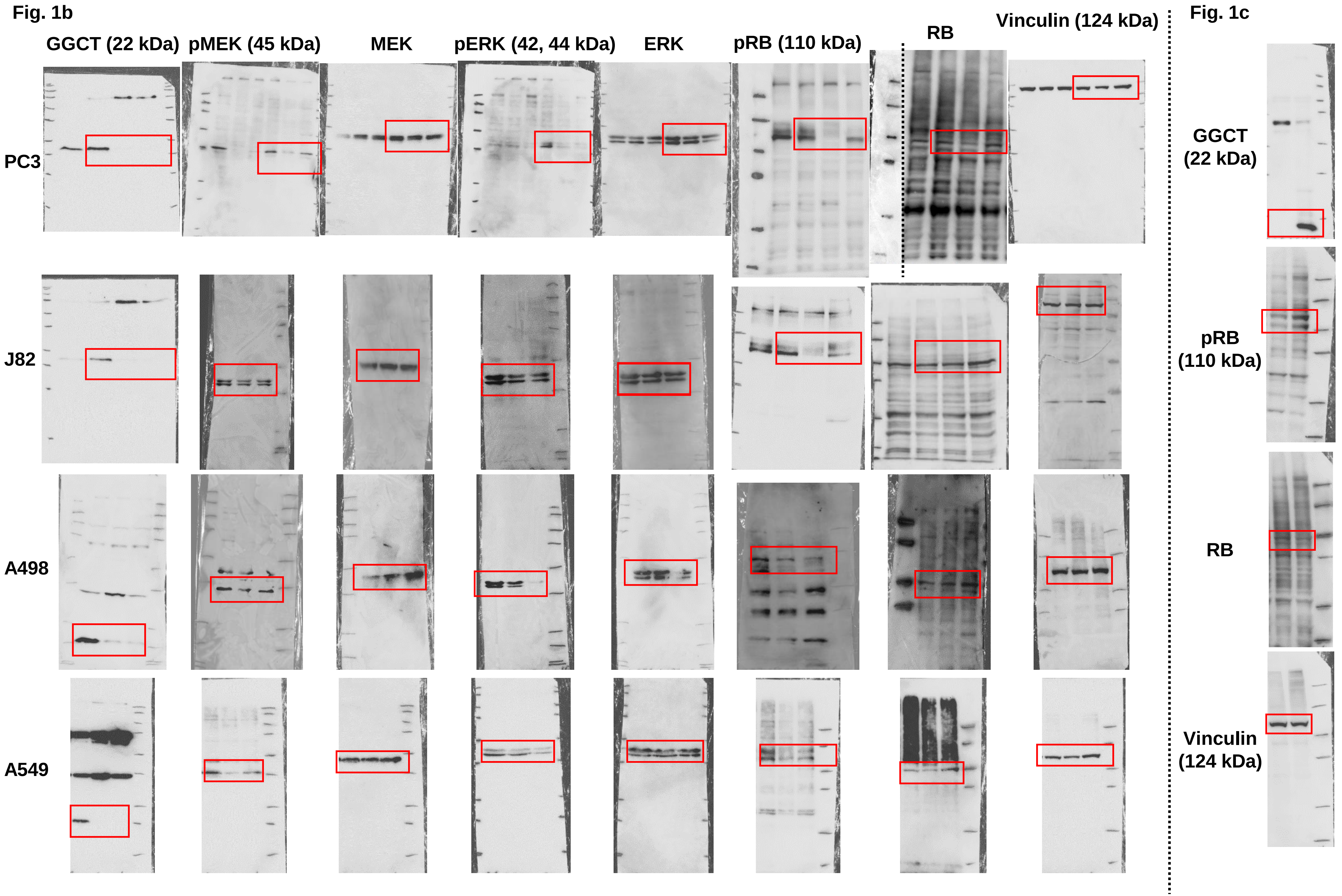

Supplementary Figure S4. Uncropped original images of Western blots in Fig. 1.

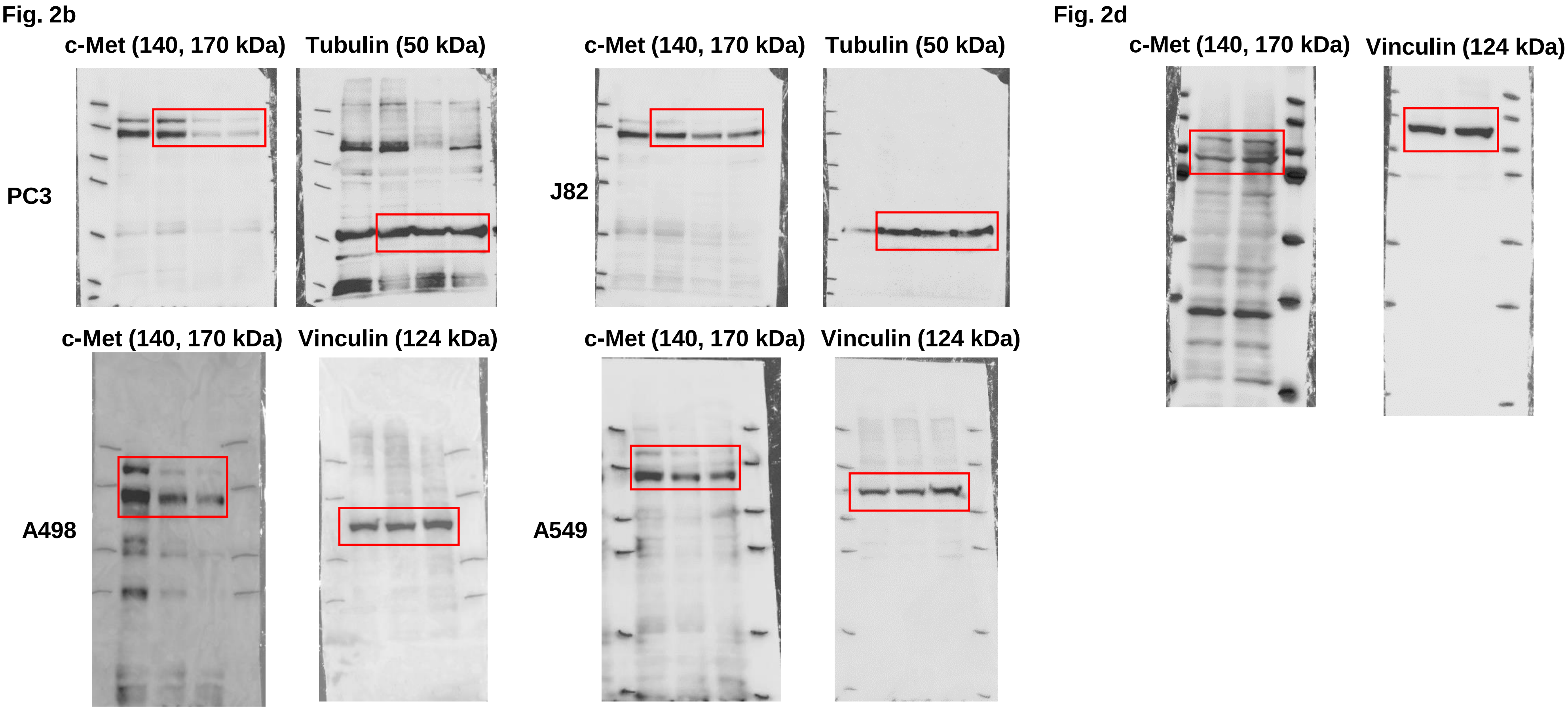

Supplementary Figure S5. Uncropped original images of Western blots in Fig. 2.

Fig. 3a

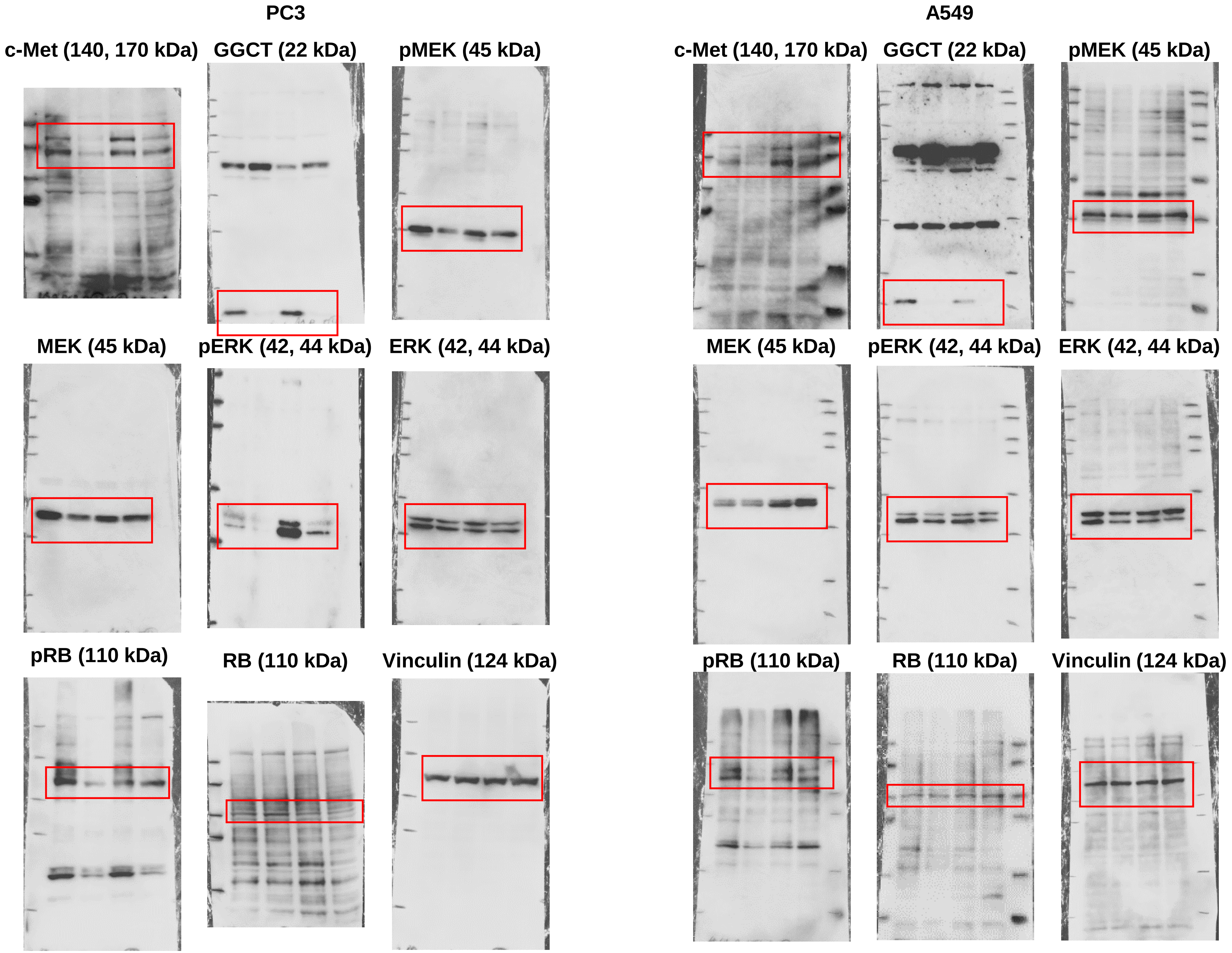

Supplementary Figure S6. Uncropped original images of Western blots in Fig. 3.

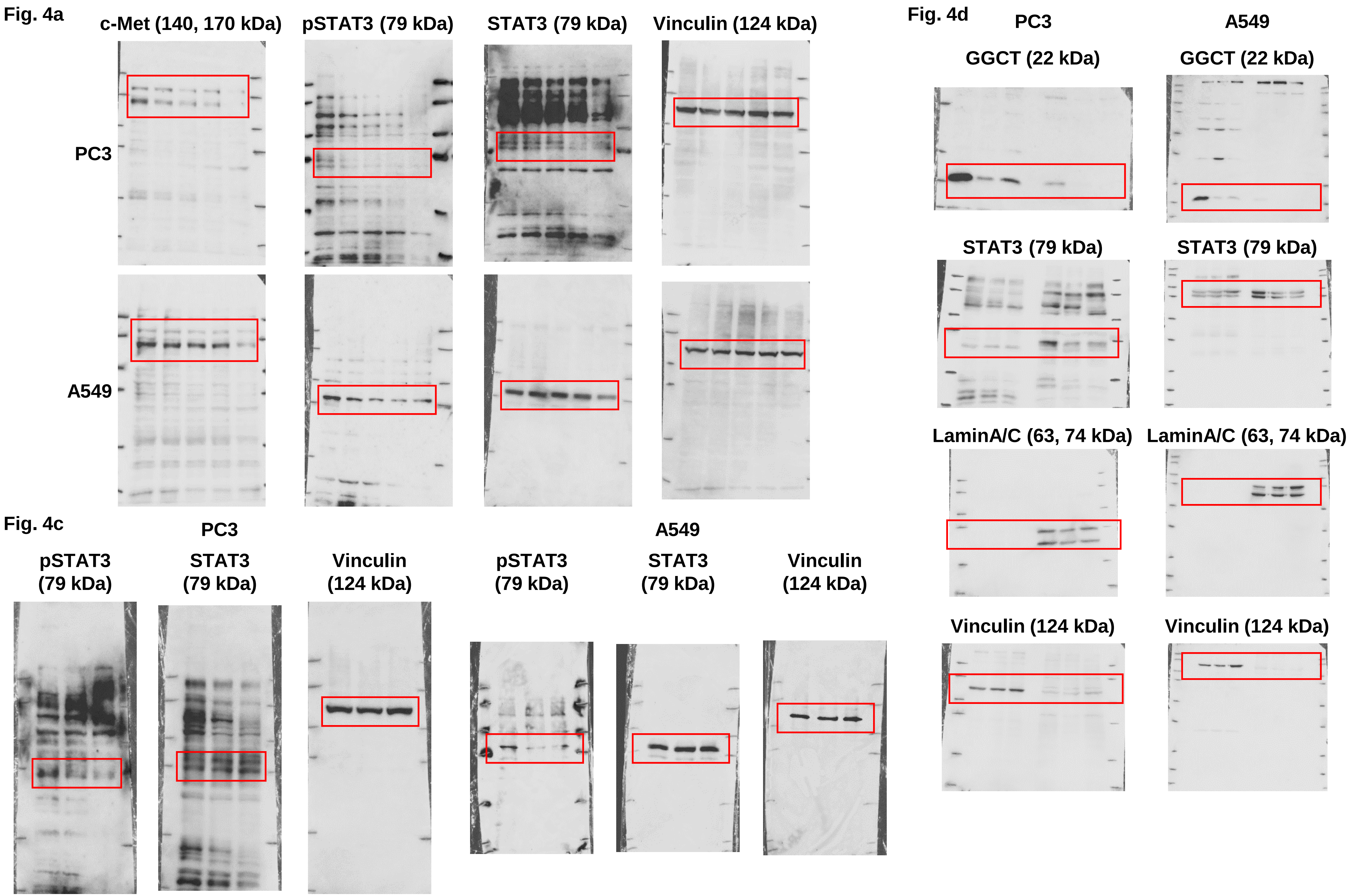

Supplementary Figure S7. Uncropped original images of Western blots in Fig. 4.

Fig. 5a

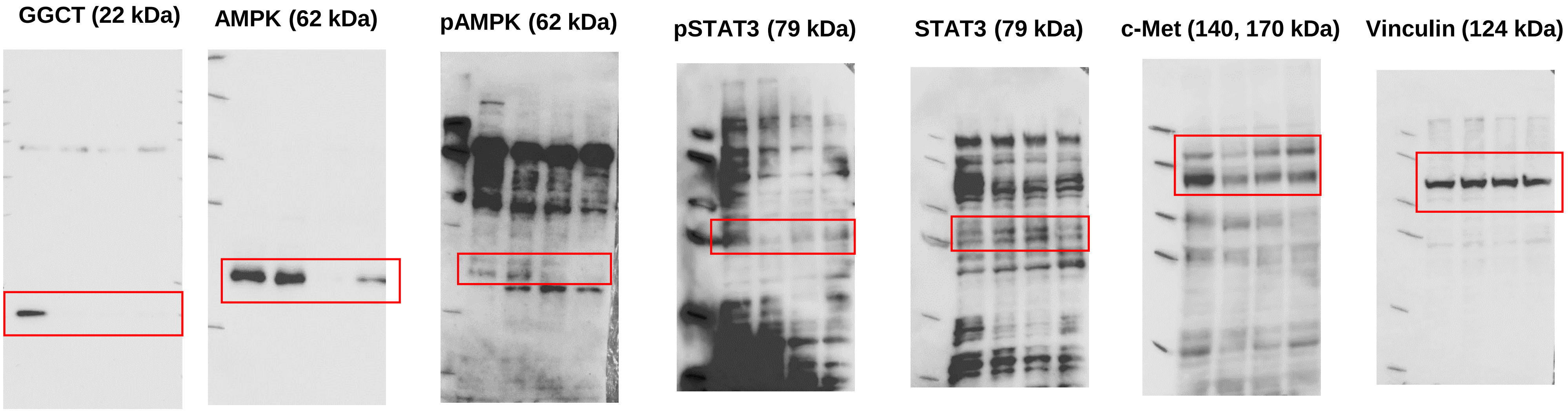

Fig. 5c

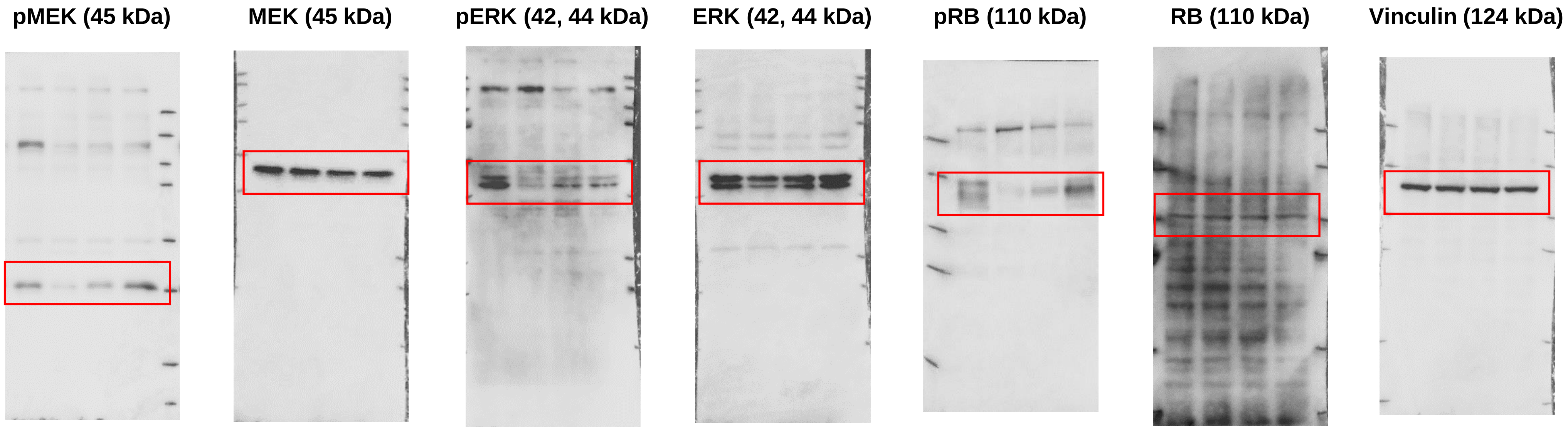

Supplementary Figure S8. Uncropped original images of Western blots in Fig. 5.
